# Novel Zinc-Attenuating Compounds as Potent Broad-Spectrum Antifungal Agents with *In Vitro* and *In Vivo* Efficacy

**DOI:** 10.1128/AAC.02024-17

**Published:** 2018-04-26

**Authors:** Karen A. O'Hanlon Cohrt, Laura Marín, Lasse Kjellerup, Johannes D. Clausen, William Dalby-Brown, José Antonio Calera, Anne-Marie Lund Winther

**Affiliations:** a Pcovery, Copenhagen, Denmark; b Instituto de Biología Funcional y Genómica, Consejo Superior de Investigaciones Científicas, Salamanca, Spain; c Department of Plant and Environmental Sciences, University of Copenhagen, Frederiksberg, Denmark; d Instituto de Biología Funcional y Genómica, Departamento de Microbiología y Genética, Universidad de Salamanca, Salamanca, Spain

**Keywords:** zinc homeostasis, antifungal, zinc deprivation, yeasts

## Abstract

An increase in the incidence of rare but hard-to-treat invasive fungal pathogens as well as resistance to the currently available antifungal drugs calls for new broad-spectrum antifungals with a novel mechanism of action. Here we report the identification and characterization of two novel zinc-attenuating compounds, ZAC307 and ZAC989, which exhibit broad-spectrum *in vitro* antifungal activity and *in vivo* efficacy in a fungal kidney burden candidiasis model. The compounds were identified serendipitously as part of a drug discovery process aimed at finding novel inhibitors of the fungal plasma membrane proton ATPase Pma1. Based on their structure, we hypothesized that they might act as zinc chelators. Indeed, both fluorescence-based affinity determination and potentiometric assays revealed these compounds, subsequently termed zinc-attenuating compounds (ZACs), to have strong affinity for zinc, and their growth inhibitory effects on Candida albicans and Aspergillus fumigatus could be inactivated by the addition of exogenous zinc to fungal growth media. We determined the ZACs to be fungistatic, with a low propensity for resistance development. Gene expression analysis suggested that the ZACs interfere negatively with the expression of genes encoding the major components of the A. fumigatus zinc uptake system, thus supporting perturbance of zinc homeostasis as the likely mode of action. With demonstrated *in vitro* and *in vivo* antifungal activity, low propensity for resistance development, and a novel mode of action, the ZACs represent a promising new class of antifungal compounds, and their advancement in a drug development program is therefore warranted.

## INTRODUCTION

The success of pathogenic microorganisms hinges upon their ability to sequester essential nutrients from their host during infection. Through a process known as nutritional immunity, the host immune system sequesters metals that are necessary for microbial growth, resulting in an extremely nutrient-limited host environment (1). For example, vertebrates express a number of iron-binding molecules, e.g., the transferrin family, that ensure extremely low concentrations of free iron in the body (2). Additionally, neutrophils and other myeloid and nonmyeloid cells synthesize large amounts of the antimicrobial Zn^2+^/Mn^2+^-chelating protein calprotectin during infection, and the contribution of calprotectin to the innate immune response against yeast and filamentous fungal pathogens is well documented (3–5).

For fungal pathogens to grow and establish infection inside their hosts, they must be able to obtain iron, zinc, and other essential metals from the harsh environment imposed by nutritional immunity (6). Consequently, successful pathogens have evolved elegant mechanisms to sequester essential metals from their hosts during infection. The mechanisms for iron sequestration are best described and include the expression of high-affinity iron transporters, iron-chelating siderophores, and iron-binding proteins (1, 7, 8). Although iron acquisition is recognized as a virulence factor for many fungal pathogens (7), research in recent years has highlighted the important contribution that zinc sequestration makes to fungal pathogenesis and virulence (4, 9). Indeed, fungal acquisition of zinc has been clearly demonstrated to be essential for fungal growth and pathogenicity, and zinc-depleting conditions are known to reduce fungal growth *in vitro* (3, 10, 11).

In all fungal species, the major zinc-binding proteins include Cu^2+^/Zn^2+^ superoxide dismutases (SODs), alcohol dehydrogenase, and ribosomal proteins (12). SODs are key enzymes in fungal virulence and are necessary for the detoxification of reactive oxygen species generated by host cells during fungal infection (13). In Aspergillus fumigatus, zinc uptake is regulated by the transcriptional regulator ZafA, and deletion of *zafA* has been shown to not only impair germination and overall growth capacity of A. fumigatus in zinc-limiting media but also completely abrogate A. fumigatus virulence in a murine model of invasive aspergillosis (11). Thus, the control of access to zinc is one of the central battlefields on which the outcome of an infection is decided. In further support of this notion, calprotectin comprises ∼40% of total protein content in the neutrophil cytoplasm during infection, and its antifungal effect can be reversed *in vitro* by micromolar quantities of zinc (3, 4, 9). Because of the great need for fungal zinc uptake during infection, it has been hypothesized that both chelation therapy and the modulation of zinc homeostasis and zinc acquisition are promising antifungal strategies (14–18).

We have previously reported the identification of novel antifungal compounds targeting the fungal plasma membrane H^+^-ATPase (19, 20). In the further optimization process a number of compounds were synthesized, and we found two of these compounds, ZAC307 and ZAC989, to be very potent inhibitors of Candida albicans growth, despite the fact that they lacked H^+^-ATPase-inhibitory activity. Due to their characteristic arrangement of an aromatic structure with nitrogen bound in close proximity to a hydroxyl group, we speculated that ZAC307 and ZAC989 could act as metal chelators. Thus, the goals of this study were (i) to investigate the chelating properties of these compounds, (ii) to characterize the spectrum of antifungal activity of these compounds *in vitro*, (iii) to ascertain whether the compounds were fungistatic or fungicidal and the propensity of C. albicans to develop resistance against these compounds, (iv) to investigate whether the antifungal activity was caused by extracellular zinc sequestration or if the compounds were taken up by Candida albicans cells, (v) to assess whether these compounds influenced the expression level of genes encoding zinc transporters required for zinc uptake from zinc-limiting media and that of other genes regulated by ZafA, which is the master regulator of zinc homeostasis in Aspergillus fumigatus, and (vi) to test and evaluate the effects of these compounds against mammalian cells and their antifungal efficacy *in vivo* in a murine model of candidiasis.

## RESULTS

### ZAC307 and ZAC989 have high binding affinity for zinc and copper but not for magnesium and calcium.

**FIG 1:**
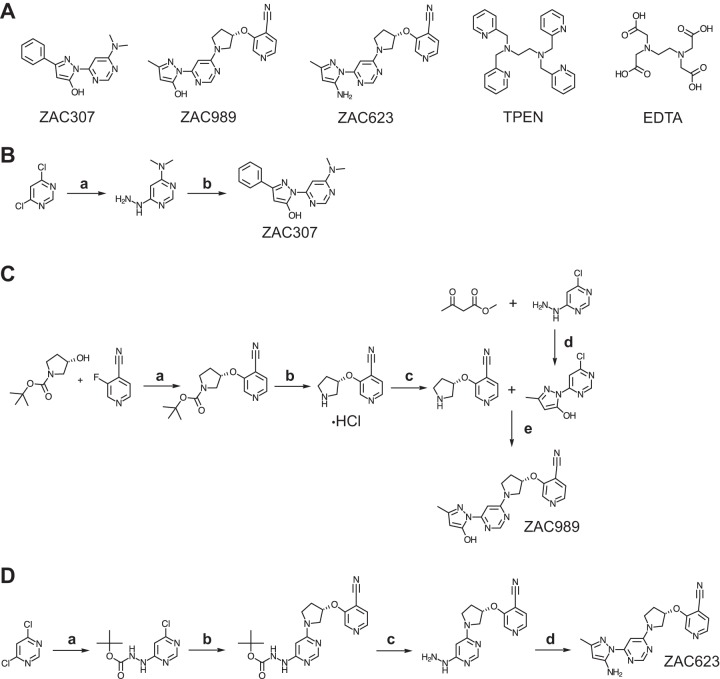
(A) Structures of the compounds ZAC307, ZAC989, ZAC623, TPEN, and EDTA. (B) Abbreviated synthetic pathway for ZAC307. (a) (i) *N*-Methylmethanamine, TEA, 2-propanol, 0°C, 2 h, evaporated; (ii) hydrazine hydrate, reflux (block temperature, 120°C), 2 h, 65%; (b) 2-propanol, reflux, 1 h, 69%. (C) Abbreviated synthetic pathway for ZAC989. (a) NaH, abs. THF, 0°C, 3 h, NH_4_Cl, 82%; (b) diethyl ether, 0°C, conc. HCl, quant.; (c) load onto SCX in methanol, elute with 1 M NH_3_ in MeOH, 95%; (d) 2-propanol, reflux 90 min, 14%; (e) NMP, DIPEA, 30 min at 100°C, 89%. (D) Abbreviated synthetic pathway for ZAC623. (a) *tert*-Butyl-*N*-aminocarbamate, DIPEA, THF, rt → reflux, 98%; (b) 3-[(3S)-pyrollidin-3-yl]oxypyridine-4-carbonitrile (see panel C reac. c), DIPEA, NMP, 120 °C, 1 h, 71%; (c) (i) TFA, DCM, rt, 30 min; (ii) load onto SCX in methanol, elute with 0.5 M NH_3_ in MeOH, quant.; (d) Z-3-amino-but-2-enenitrile, AcOH, EtOH, 80°C 4 h, 85%.

ZAC307, ZAC989, ZAC623 (collectively referred to as ZACs), and the reference compounds EDTA and TPEN [N,N,N’,N’-tetrakis(2-pyridylmethyl)-ethylenediamine] (Fig. 1) were evaluated for their zinc binding properties. ZAC307 and ZAC989 have dissociation constants (*K_d_*) in the low nanomolar range (13 to 71 nM), as determined by a fluorescence-based competition assay (Table 1). ZAC623 exhibited poor affinity for zinc, with a dissociation constant of >6 μM. Dissociation constants for EDTA and TPEN could not be determined with this assay, as they were below the measurable range, but both have previously been reported to be very potent zinc chelators (21).

**TABLE 1:** Dissociation constant determination between chelating compounds and zinc^*a*^

Compound	Compound-Zn^2+^ *K*_D_ (μM)
ZAC989	0.013
ZAC307	0.071
ZAC623	>6
EDTA	<0.01
TPEN	<0.01

a *K_d_* determination for the compound-Zn^2+^ complex was performed with a fluorescence-based competition assay using FluoZin-3.

**FIG 2:**
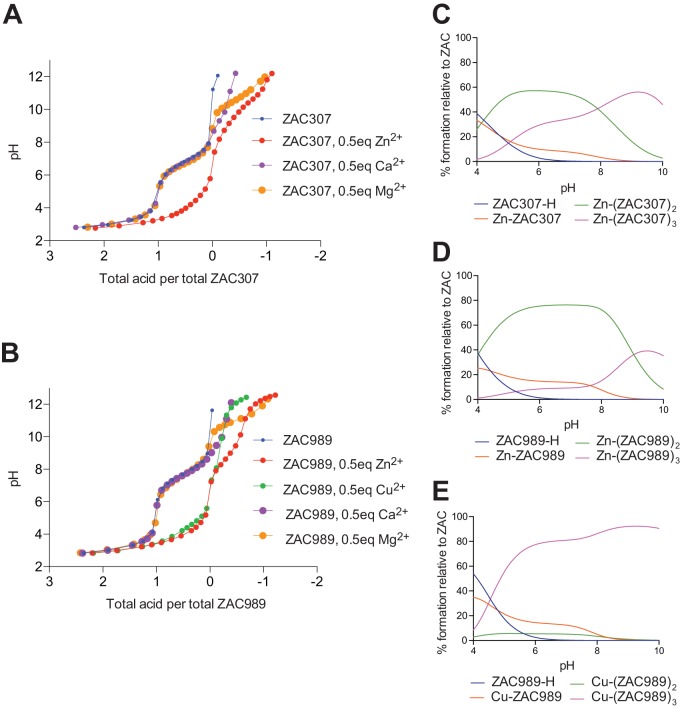
Potentiometric titration results for ZAC307 and ZAC989. (A) Potentiometric equilibrium curves of ZAC307 in the absence or presence of 0.5 mol equivalent of Zn(NO_3_)_2_, CaCl_2_, or MgCl_2_ in DMSO-water (70:30, vol/vol). (B) Potentiometric equilibrium curves of ZAC989 in the absence or presence of 0.5 mol equivalent of Zn(CF_3_SO_3_)_2_, CuSO_4_, CaCl_2_, or MgCl_2_ in DMSO-water (70:30, vol/vol). (C, D, and E) Species distribution diagram as a function of pH for a system containing 0.5 mM Zn(II) and 1 mM ZAC307 (C), 0.5 mM Zn(II) and 1 mM ZAC989 (D), and 0.5 mM Cu(II) and 1 mM ZAC989 (E).

The Zn^2+^-binding properties of ZAC307 and ZAC989 were further evaluated using a potentiometric assay, where pH is measured as a function of base (NaOH) added to the compound in either the absence or presence of metal. Since potentiometric methods require millimolar concentrations, and ZAC307 and ZAC989 displayed poor solubility in water at such high concentrations, the measurements were performed in a mixture of dimethyl sulfoxide (DMSO) and water (70:30, vol/vol), as described previously (22). To determine the deprotonation constant, a solution of 1 mM ZAC307 or ZAC989 was titrated with 0.3 M NaOH at constant ionic strength (Fig. 2A and B). In a second run, the same titration was performed in the presence of 0.5 eq of Zn^2+^ for ZAC307 and ZAC989. A shift in the pH curve in the presence of the metal (ZAC307, 0.5 eq of Zn^2+^ [Fig. 2A]) compared to the absence of the metal (ZAC307 [Fig. 2A]) indicates binding of Zn^2+^ to the compound. The measured pH data were analyzed with the Hyperquad program suite, taking into account all relevant equilibrium constants, including also the constants for metal hydroxylation. The analysis provides the pK_a_ values and metal complex stability constants, as well as ligand-metal complex speciation calculation, indicating how many ZAC molecules are involved in coordinating the Zn^2+^ ion at different pH values. Refinement of the measured pH data for ZAC307 provided a pK_a_ value of 6.84 and formation constants log β_1_, log β_2_, and log β_3_ of 7.47, 13.27, and 18.14, corresponding to the formation of 1:1, 2:1, and 3:1 ligand-Zn(II) complexes, respectively. The corresponding species distribution diagram is displayed in Fig. 2C, and it shows that at neutral pH in a DMSO-water solvent mixture, the ligand-zinc stoichiometry is a mixture of 1:1, 2:1, and 3:1 binding, with 2:1 and 3:1 being the dominant stoichiometries. Similar potentiometric experiments were carried out with CaCl_2_ and MgCl_2_ in place of Zn(NO_3_)_2_, and these revealed that Ca^2+^ and Mg^2+^ binding to ZAC307 is negligible (Fig. 2A). Potentiometric titration of ZAC307 with CuSO_4_ was not possible due to precipitation of the resulting complex, suggesting that ZAC307 also binds copper. Refinement of the measured pH data for ZAC989 provided a pK_a_ value of 7.70, and in the presence of zinc we measured formation constants log β_1_, log β_2_, and log β_3_ of 7.35, 14.30, and 19.71, corresponding to the formation of 1:1, 2:1, and 3:1 ligand-Zn(II) complexes, respectively (Fig. 2D). Potentiometric experiments were also carried out with ZAC989 and CuSO_4_ and with CaCl_2_ and MgCl_2_, and the data indicated that ZAC989 chelates copper, whereas binding of calcium and magnesium is negligible. ZAC989 bound copper with the formation constants log β_1_, log β_2_, and log β_3_ of 7.83, 13.37, and 20.62, respectively. The most dominant ZAC989-copper stoichiometry was a 3:1 stoichiometry (Fig. 2E).

### ZACs are potent broad-spectrum fungistatic yeast inhibitors that work intracellularly and display low potential for resistance development.

**FIG 3:**
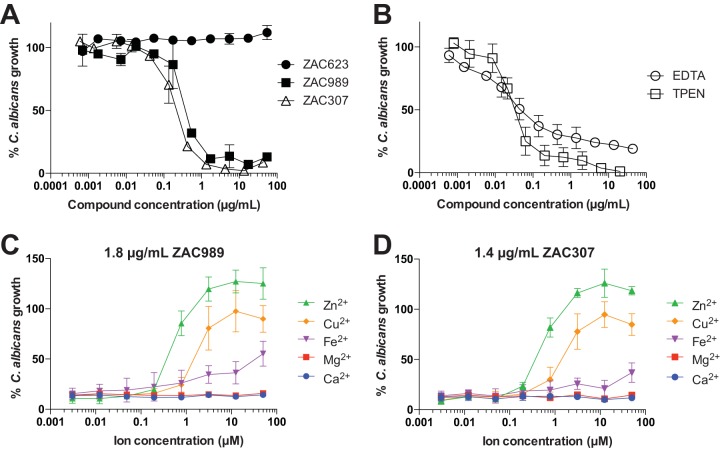
(A) Candida albicans growth inhibition by ZAC989 and ZAC307 but not by the closely analogous compound ZAC623. (B) Candida albicans growth inhibition by EDTA and TPEN. (C and D) Abrogation of the antifungal effect of ZAC989 and ZAC307 in Candida albicans cells was achieved by the addition of Zn^2+^ or Cu^2+^ ions. The graphs display how much Zn^2+^, Cu^2+^, Fe^2+^, Mg^2+^, or Ca^2+^ is required to abrogate the antifungal effects of 1.8 μg/ml of ZAC989 or 1.4 μg/ml of ZAC307. C. albicans growth (expressed as percent) in panels A to D was normalized to C. albicans growth in RPMI medium containing 1.5% DMSO. Graphs show means ± SEMs for 2 or 3 independent experiments.

ZAC307 and ZAC989 exhibit antifungal activity and display potent growth inhibition in the low microgram-per-milliliter range (0.2 to 0.9 μg/ml) against a number of pathogenic Candida species, including a Candida glabrata strain with increased efflux pump activity (Table 2). The MIC was defined as the lowest compound concentration that resulted in at least 50% growth inhibition for yeasts, which corresponded to a prominent decrease in visible growth. For molds the MIC was defined as the lowest concentration of the compound that resulted in no visible growth. In Candida albicans this value was 0.6 μg/ml for ZAC989 and 0.4 μg/ml for ZAC989. ZAC623 did not display growth-inhibitory activity against C. albicans (Fig. 3A). The known potent metal chelators EDTA and TPEN both exhibited a MIC of ∼0.05 μg/ml, but TPEN led to a more complete growth inhibition than did EDTA (Fig. 3B). The antifungal effects of ZAC989 and ZAC307 were reversed by exogenous addition of zinc or copper to the growth medium in the presence of either ZAC989 or ZAC307 (Fig. 3C and D). Zinc ions were most effective in reversing the growth-inhibitory effects of the ZACs, with restoration of fungal growth observed in the presence of 1 μM Zn^2+^. Addition of iron (Fe^2+^) had a modest effect on the antifungal activity of ZAC989 and ZAC307, with a fungal growth rate of approximately 50% in the presence of 100 μM Fe^2+^ compared to control cells. In accordance with the results obtained from potentiometric titration, the addition of magnesium or calcium had no effect on the antifungal activity of the ZACs (Fig. 3C and D).

**TABLE 2:** MICs for several different Candida species

Compound	MIC (μg/ml) for Candida isolate					
	C. albicans SC5314	C. parapsilosis ATCC 22019	C. glabrata ATCC 90030	C. glabrata Cg003^*a*^	C. tropicalis Ct016	C. krusei ATCC 6258
ZAC989	0.6	0.8	0.9	0.6	0.9	0.8
ZAC307	0.4	0.4	0.2	0.2	0.4	0.4
ZAC623	>54	>54	ND^*b*^	ND	ND	>54

a This strain has mutations resulting in increased efflux pump activity compared to that of wild-type isolates (34).

b ND, not determined.

**FIG 4:**
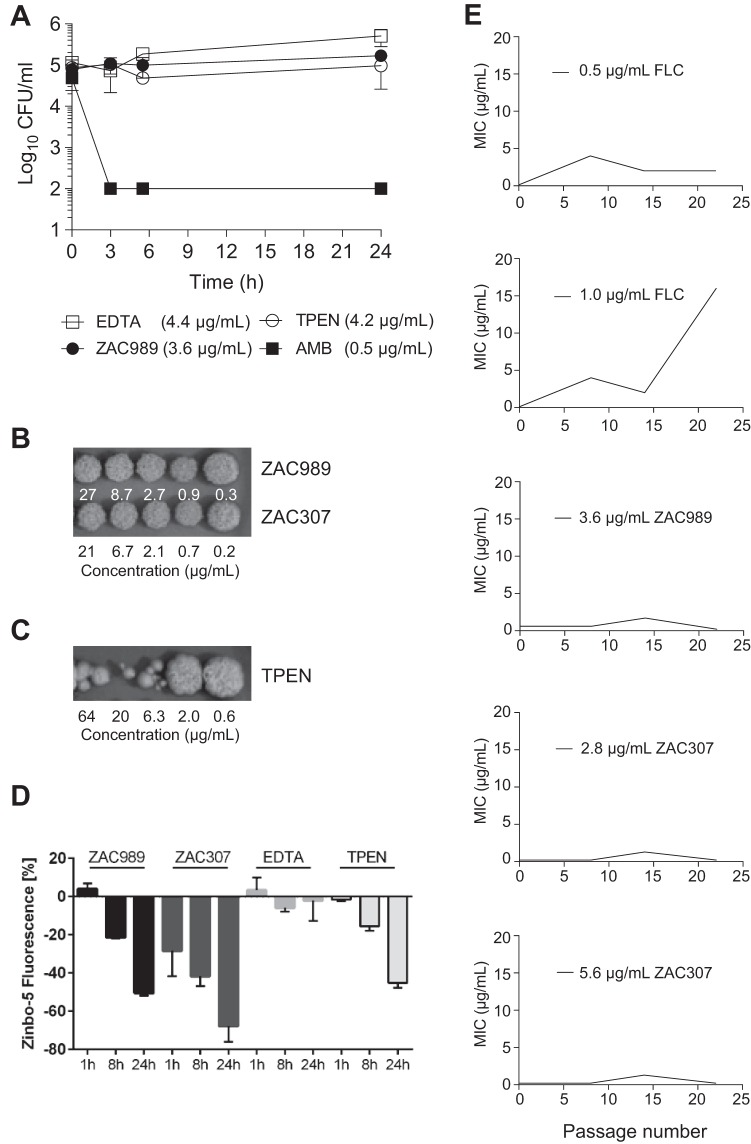
(A) Time-kill experiments with C. albicans revealed that ZACs exhibit fungistatic activity. The following final concentrations of compound were applied: 0.5 μg/ml of amphotericin B (AMB), 3.6 μg/ml of ZAC989, 4.4 μg/ml of EDTA, and 4.2 μg/ml of TPEN. Data are means ± SEMs for two biological replicates. (B and C) C. albicans cells exposed to ZAC989 and ZAC307 for 5 days resume normal growth (B), while cells exposed to TPEN for 5 days show poor recovery (C). C. albicans cells exposed to EDTA resumed visible growth after 48 h of compound incubation (concentration range, 0.22 μg/ml to 22 μg/ml) and were therefore not evaluated for MFC. (D) A decrease in intracellular zinc as evidenced by a decrease in Zinbo-5 fluorescence signal suggests that ZAC989 and ZAC307 act intracellularly in C. albicans. The following final concentrations of compound were applied: 9 μg/ml of ZAC989, 7 μg/ml of ZAC307, 15 μg/ml of EDTA, and 1.3 μg/ml of TPEN. Data are means ± SEMs for two biological replicates. (E) Resistance induction study. There was no change in the MIC for the ZACs after repeated ZAC exposure, while a significant increase in the MIC was observed for C. albicans after 22 passages with repeated exposure to fluconazole (FLC) (1.0 μg/ml).

Time-kill investigations revealed that the ZACs exhibited fungistatic activity against C. albicans, in contrast to amphotericin B (AMB), which exhibits fungicidal activity after 3 h of exposure (Fig. 4A). Both EDTA and TPEN exhibited a fungistatic effect within the first 24 h of exposure. Fungal growth recovery was evaluated after longer times of exposure of C. albicans cells to the ZACs. This revealed that the fungal cells were able to resume growth when moved to fresh growth media in the absence of ZACs (Fig. 4B). Fungal cells exposed to TPEN at concentrations above 2 μg/ml showed poor recovery, and this may be explained by the strong chelating properties of TPEN that enable it to extract zinc from essential enzymes, leading to fungal cell death after prolonged exposure (Fig. 4C).

In order to gain an understanding of the potential of ZACs to permeabilize into fungal cells, we monitored the intracellular zinc levels of C. albicans cells that were exposed to TPEN, EDTA or ZACs using the cell-permeable fluorescent probe Zinbo-5. The affinity constant of this probe for zinc is 2.2 nM (23), which is weaker than that of most zinc-binding proteins, and thus it reports only the free or weakly bound zinc ions. The probe localizes to the internal membrane system, including the endoplasmic reticulum (ER), in C. albicans (L. Kjellerup, A. L. Winther, D. Wilson, and A. T. Fuglsang, submitted for publication). A decreased Zinbo-5 fluorescent signal in the presence of fungal cells, compound, and zinc would indicate that intracellular Zinbo-5 is competing with the added compound for zinc ions. ZAC307 and ZAC989 were evaluated at a concentration of 25 μM, equivalent to 7 μg/ml and 9 μg/ml, respectively. ZAC989 induced a time-dependent decrease in Zinbo-5 fluorescence, similarly to 1.3 μg/ml of TPEN (Fig. 4D). ZAC307 decreased the Zinbo-5 fluorescence after only 1 h of incubation, and this decrease was greater than that observed for ZAC989 (Fig. 4D), despite ZAC307 having a 5-fold-lower affinity for zinc than ZAC989. These data suggested that ZAC307, ZAC989, and TPEN were cell permeative and bound intracellular zinc. In agreement with this, the extracellular chelator EDTA did not reduce the Zinbo-5 fluorescence under the same conditions.

We investigated the propensity for development of resistance to ZAC307 and ZAC989 by repeated exposure of C. albicans to ZACs in SDwoz medium (see Materials and Methods) over a 36-day period. We observed no change in the MIC for the ZACs after repeated ZAC exposure. In contrast, cells repeatedly exposed to fluconazole exhibited a significant increase in the MIC for fluconazole after 22 passages (Fig. 4E). Based on these results, it appeared that ZAC resistance was not easily induced in C. albicans.

### ZACs efficiently inhibit the growth capacity of Aspergillus fumigatus under zinc-limiting conditions, and their inhibitory effects are inactivated by zinc.

**FIG 5:**
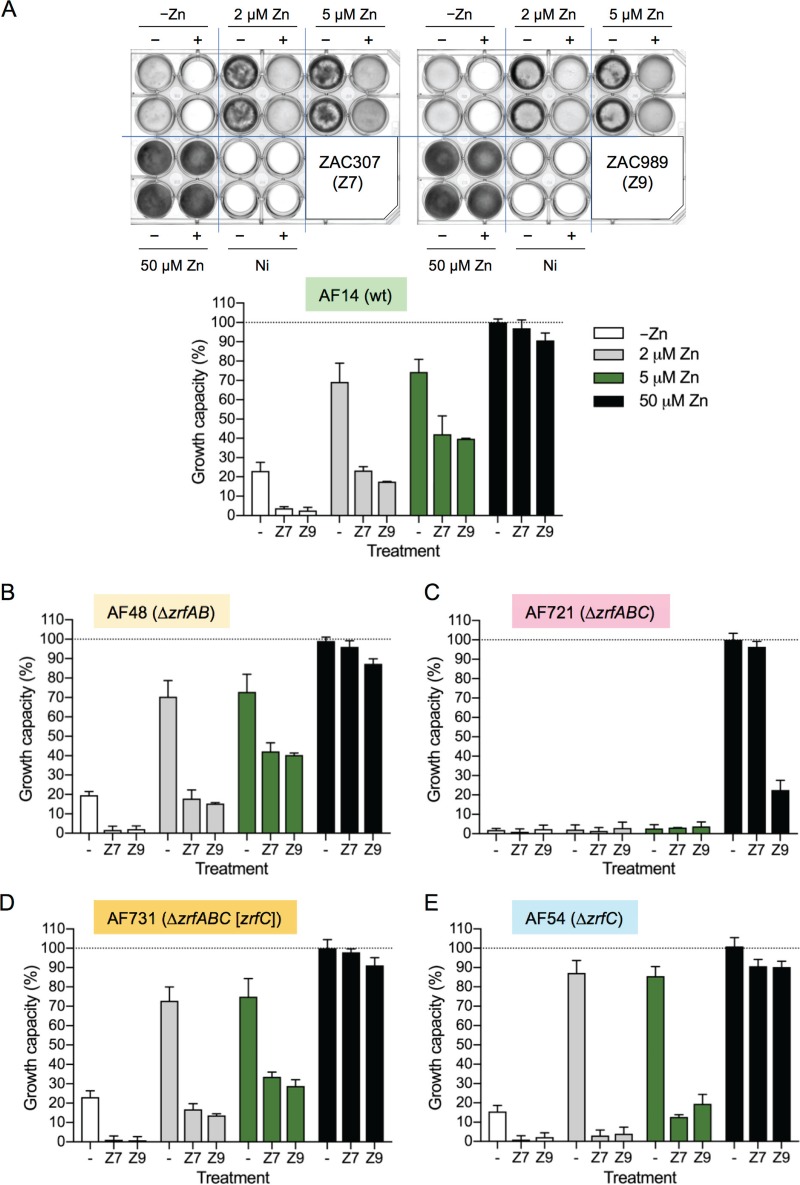
Effects of ZAC307 and ZAC989 on the growth capacity of several A. fumigatus strains. (A) The wild-type AF14 strain was cultured in 24-well culture plates inoculated with 10^5^ conidia per well in a total volume of culture medium of 1 ml. Culture media that were not inoculated (Ni) were used as background reference. Media were not supplemented with zinc or were supplemented with 2, 5, or 50 μM ZnSO_4_ in the absence (−) or the presence (+) of ZAC307 (Z7) or ZAC989 (Z9), as indicated. Plates were incubated at 37°C in a humid atmosphere for 44 h, scanned, and quantified, and the growth was represented graphically. (B) Effect of ZACs on the growth capacity of the Δ*zrfA* Δ*zrfB* mutant strain (AF48). (C) Effect of ZACs on the growth capacity of the Δ*zrfA* Δ*zrfB* Δ*zrfC* mutant strain (AF721). (D) Effect of ZACs on the growth capacity of the Δ*zrfA* Δ*zrfB* Δ*zrfC* (*zrfC*) mutant strain (AF731). (E) Effect of ZACs on the growth capacity of the Δ*zrfC* mutant strain (AF54). The AF48, AF721, AF731, and AF54 strains were all cultured and incubated in 24-well culture plates, and their growth was quantified as described for the wild-type strain. In all cases the relative arbitrary units obtained after quantification of the plates were normalized by taking the average of the background values of cultures which were not inoculated as a growth capacity of 0% and the growth reached by the wild-type strain in media supplemented with 50 μM zinc in the absence of ZACs as a growth capacity of 100%. In all graphs, the data represent the averages and SDs from two independent experiments in which all strains had been cultivated in duplicate.

In addition to potent antifungal effects on the five Candida species tested, ZAC307 and ZAC989 also potently inhibited the mold Aspergillus fumigatus and other Aspergillus species, as well a number of rare but very hard-to-treat members of the Mucorales order, including *Rhizopus oryzae*, Rhizopus microsporus, and *Mucor indicus*. The ZACs inhibited these molds and mucorales isolates in a range from 0.4 μg/ml to 5.4 μg/ml (Table 3). To assess the capacity of the ZACs to inhibit A. fumigatus growth in the presence of zinc, 1-ml aliquots of sRPMI zinc-limiting medium (RPMI 1640 medium [R8755; Sigma] supplemented with 10 μM FeSO_4_·7H_2_O, 1 μM CuSO_4_·5H_2_O, and 1 μM MnCl_2_·H_2_O) or this medium supplemented with 2, 5, or 50 μM zinc were inoculated with 10^5^ conidia of a wild-type A. fumigatus strain (AF14), dispensed in 24-well culture plates, and incubated in the presence of either ZAC307 or ZAC989 at a final concentration 21 μg/ml or 27 μg/ml (equivalent to 75 μM), respectively (Fig. 5A). Graphical representation and quantification of the fungal growth in 24-well culture plates in the presence of ZACs (Fig. 5A) revealed that the growth capacity of a wild-type A. fumigatus strain was reduced under zinc-limiting conditions but increased gradually when the growth medium was supplemented with increasing amounts of zinc, until fungal growth was fully restored when cultured in medium supplemented with 50 μM Zn (i.e., under zinc-replete conditions). Hence, the inhibitory effects of the ZACs against A. fumigatus were completely counteracted by simultaneous addition of zinc, similar to our observations for C. albicans (Fig. 3C and D).

**TABLE 3:** MICs for several different Aspergillus and Mucorales species

Compound	MIC (μg/ml) for isolate					
	Aspergillus fumigatus ATCC 13073	Aspergillus flavus ATCC 15547	Aspergillus terreus At070	*Rhizopus oryzae* ATCC 34965	Rhizopus microsporus ATCC 66276	*Mucor indicus* ATCC MYA-4678
ZAC989	5.4	5.1	1.6	1.7	0.5	1.1
ZAC307	4.0	1.3	1.3	1.3	0.4	1.3

### The zinc transporter ZrfC plays an important role in regulating fungal sensitivity to the ZACs.

To ascertain whether ZAC307 and ZAC989 interfered with zinc uptake from the sRPMI zinc-limiting medium, we analyzed their effects on the growth capacity of the mutant strains AF48 (Δ*zrfA* Δ*zrfB*), AF721 (Δ*zrfA* Δ*zrfB* Δ*zrfC*), AF731 (Δ*zrfA* Δ*zrfB* Δ*zrfC* [*zrfC*]), which is an AF721 derivative strain that carries the *zrfC* gene reintroduced at the *pyrG* locus as described previously (24), and AF54 (Δ*zrfC*) (Fig. 5B to E). The overall effect of the ZACs on the growth capacity of the AF48 strain was similar to that of the wild-type strain AF14 (compare Fig. 5A and B). In the absence of ZACs, both strains exhibited a reduced growth capacity, from 100% to 70%, when cultured in media supplemented with 50 μM compared to media supplemented with 2 μM zinc. This corresponded to a 1.4-fold reduction in growth capacity. However, in the presence of 2 μM zinc plus 75 μM ZAC307, the growth capacity of AF14 and AF48 was reduced, respectively, 2.9- and 3.9-fold compared to that in the presence of 50 μM zinc. Similarly, 2 μM zinc plus 75 μM ZAC989 reduced the growth capacity of AF14 and AF48 3.9- and 4.5-fold, respectively. The AF721 strain did not grow under zinc-limiting conditions, and hence the effect of these compounds on this strain could not be tested (Fig. 5C). The reintroduction of *zrfC* in a strain with a Δ*zrfA* Δ*zrfB* Δ*zrfC* genetic background restored the fungal growth capacity in the presence of the ZACs (Fig. 5D) at the same level as that of the wild-type and AF48 strains (Fig. 5A and B). Finally, in the presence of 2 μM zinc either with or without simultaneous exposure to ZAC307 or ZAC989, the growth capacity of the AF54 strain was reduced an average of 24.6-fold compared to its growth capacity in the presence of 50 μM zinc (Fig. 5E); i.e., the ZACs were between 5- and 8-fold more efficient as inhibitors of the growth capacity of a Δ*zrfC* strain than of a wild-type or Δ*zrfA* Δ*zrfB* strain, which suggested that the effect of ZACs could be counteracted to a certain extent by the function of ZrfC.

### ZAC307 and ZAC989 inhibit the transcription of genes regulated by ZafA under zinc-limiting conditions.

The major regulator of the A. fumigatus zinc homeostatic response under zinc-limiting conditions is the transcription factor ZafA (11), which is a zinc-responsive factor that senses the intracellular concentration of zinc in a way similar to that of its orthologue Zap1 in the yeast Saccharomyces cerevisiae (25). Thus, when the cytoplasmic zinc content is high enough, ZafA becomes saturated with Zn^2+^ ions and adopts a transcriptionally inactive conformation. In contrast, when the intracellular concentration drops below a certain threshold, ZafA begins to release Zn^2+^ ions and gradually adopts a transcriptionally active conformation, whereby it is able to induce the expression of *zrfA* and *zrfB* in acidic zinc-limiting media and of *zrfC* in alkaline zinc-limiting media (11, 24).

**FIG 6:**
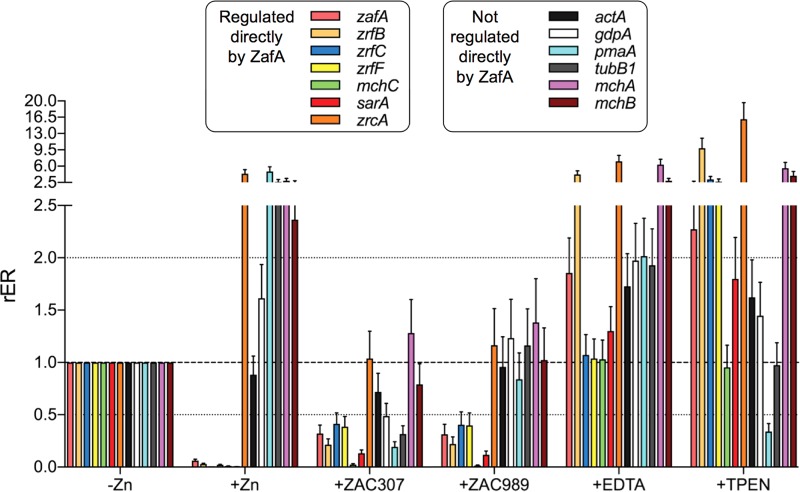
Effects of the ZACs, the extracellular chelator EDTA, and the intracellular chelator TPEN on the transcription of ZafA target genes under zinc-limiting conditions. The wild-type strain precultured in the sRPMI zinc-limiting medium for 20 h at 37°C with shaking at 200 rpm was left untreated (−Zn) or treated with 20 μM zinc (+Zn), 21 μg/ml of ZAC307 (+ZAC307), 27 μg/ml of ZAC989 (+ZAC989), 146 μg/ml of EDTA (+EDTA), and 10.6 μg/ml TPEN (+TPEN). The expression level of the indicated genes was analyzed by RT-qPCR using 18S rRNA as an internal reference. The changes in the relative expression ratios (rER) were measured after 2 h of incubation following treatment with the different compounds and compared to the expression levels observed under zinc-limiting conditions (−Zn). The bar diagram depicts the averages and SDs of the results obtained in two independent experiments.

We employed quantitative reverse transcription-PCR (RT-qPCR) to assess whether exposure to ZAC307, ZAC989, EDTA, or TPEN influenced the expression of several ZafA target genes and other genes not regulated directly by ZafA (as controls) (Table 4). The set of genes investigated was selected based on a genome-wide transcription analysis of A. fumigatus grown under zinc-limiting conditions that had been performed previously in our laboratory (J. A. Calera, unpublished data), using the primers listed in Table 5. All selected ZafA target genes were induced by ZafA under zinc-limiting conditions with the exception of the putative zinc storage vacuole transporter *zrcA*, which was repressed by ZafA under zinc-limiting conditions (Calera, unpublished). As expected, the relative expression levels of all ZafA target genes induced by ZafA under zinc-limiting conditions were dramatically reduced, to almost undetectable levels, upon the addition of Zn^2+^ (Fig. 6). In contrast, the expression level of the *zrcA* gene, which was repressed by ZafA, and most of the genes not regulated by ZafA increased under zinc-replete conditions to different extents, with the exception of *actA*, whose expression level remained similar to that observed before the zinc shock. Interestingly, exposure to either ZAC307 or ZAC989 inhibited the expression of the ZafA target genes similarly. We also observed reduced expression levels for most of the investigated genes that were not regulated by ZafA following ZAC307 exposure. In particular, the expression level of *pmaA*, which encodes the orthologue of the Pma1 H^+^-ATPase from S. cerevisiae, and that of the *gdpA* and *tubB1* genes were reduced at levels similar to that of the ZafA target genes (Fig. 6). In contrast, treatment with ZAC989 did not have a noticeable effect on the expression levels of the genes not regulated by ZafA, which remained similar to that observed under zinc-limiting conditions (Fig. 6). This finding suggested that although the overall outcomes of the treatment with ZAC307 and ZAC989 on ZafA-regulated genes were quite similar, the precise mode of action of ZAC307 on gene expression was different from that of ZAC989, which appeared to inhibit the ZafA regulated genes more specifically than ZAC307.

**TABLE 4:** Selected genes for quantifying relative expression level by RT-qPCR

Regulation by ZafA	Gene	Code	Function
Regulated	*zafA*	AFUA_1G10080	Major transcriptional regulator of zinc homeostasis
	*zrfB*	AFUA_2G03860	Zinc transporter of the ZIP family putatively located in the cytoplasmic membrane
	*zrfC*	AFUA_4G09560	Zinc transporter of the ZIP family putatively located in the cytoplasmic membrane
	*zrfF*	AFUA_2G08740	Zinc transporter of the ZIP family putatively located in vacuolar membrane
	*zrcA*	AFUA_7G06570	Zinc transporter of the CDF family putatively located in vacuolar membrane
	*mchC*	AFUA_8G02620	Putative zinc metallochaperone
	*sarA*	AFUA_7G06810	Putative l-amino acid oxidase
Not regulated	*actA*	AFUA_6G04740	Actin
	*tubB1*	AFUA_1G10910	β-Tubulin subunit 1
	*gdpA*	AFUA_5G01970	Glyceraldehyde-3-phosphate dehydrogenase
	*pmaA*	AFUA_3G07640	Plasma membrane H^+^-ATPase
	*mchA*	AFUA_2G11720	Putative metallochaperone
	*mchB*	AFUA_4G07990	Putative metallochaperone

**TABLE 5:** Primers used to quantify mRNA by RT-qPCR

Oligonucleotide	Sequence (5′ → 3′)
18SRNA-D	TGTTAAACCCTGTCGTGCTG
18SRNA-R	GTACAAAGGGCAGGGACGTA
ZAFA- D1	GGCAAGTCATTTACCGACAGC
ZAFA-R1	TCGATGACTTGACATGTTGGACG
ZRFB-D	ACCGGCAGAAGAAGCATTGA
ZRFB-R	ACCGCATCACCATCAACTCA
ZRFC-D	CAAACTCTCGGTGCTCGTCA
ZRFC-R	GAAGACAATCACCACCAGCA
qZRFF2-D	CGTATTCCCTCTCATGTCGTCG
qZRFF2-R	AGAGCCATTTGCCTGGTTCG
SARA-D	GCATATCATGTCACCGAGCACA
SARA-R	AGCCCCAACTCCAACAACAA
qMCHC-D	CATGCTAACGATGGGATGCG
qMCHC-R	CTTCGGTCTCCCAATGGTGG
qZRCA-D	TGCAGAGTGTTCCTCTCGGAGTCG
qZRCA-R	TCGCCAGATATGCAGTTCATGGACG
qACT3-D	CCACGTCACCACTTTCAACTCCATC
qACT3-R	TCCTTCTGCATACGGTCGGAGATAC
qGDPA2-D	CTCACTTGAAGGGTGGTGCC
qGDPA2-R	GATGTCGGAGGTGTAGGTGG
qPMA12-D	AGATCGCTACTCCTGAGCACG
qPMA12-R	CTTCTGCTCGGCAAGGTAAGC
BTUB-D	AACAACATCCAGACCGCTCT
BTUB-R	TGATCACCGACACGCTTGAA
qMCHA-D	GAAACCGCAACGAGCCATAC
qMCHA-R	ACGAGATCCGCCTTGTTCAG
qMCHB-D	TGATCTTGAGGTGCAGACGC
qMCHB-R	TGATGGTCATCCGTCAACCG

Finally, we anticipated that chelation of extracellular zinc upon addition of a relatively high concentration of EDTA to the culture media for a short period (2 h) would result in a transient hyperactivation of ZafA and concomitant upregulation of the most direct ZafA target genes, including those encoding zinc transporters. We expected to observe the same effect with TPEN treatment, although in this case chelation of intracellular zinc should exacerbate the zinc starvation status of the fungal cells, leading to a more extended hyperactivation of ZafA and higher expression of the ZafA target genes than attained with EDTA after the same incubation period. The expression profile for the ZafA-regulated genes observed in EDTA- or TPEN-treated cultures reflected precisely what we predicted (Fig. 6).

In summary, these results suggested that the antifungal effects of ZAC307 and ZAC989 were most likely mediated through a mechanism that ultimately results in the inhibition of the transcriptional activation activity of ZafA.

### Cytotoxicity and off-target activity studies.

ZAC307, ZAC989, EDTA, and TPEN were evaluated for mammalian cytotoxicity in a standard hepatocyte proliferation assay, where the mammalian HepG2 cell line was exposed to the compounds for either 24 h or 72 h. After 24 h of exposure to ZAC307 and ZAC989, the half-maximal effective concentration (EC_50_) was >28 μg/ml, while after 72 h of exposure, the EC_50_s were 13.2 μg/ml and 6.9 μg/ml, respectively (Table 6). With antifungal activity against yeast and the Mucorales isolates in the 0.2- to 1.7-μg/ml range, the compounds exhibited a reasonable selectivity index toward mammalian cells. However, the selectivity index between the Aspergillus species and mammalian cells was limited. The nonpermeative chelator EDTA did not affect the proliferation of HepG2 cells, while the potent zinc chelator TPEN had an EC_50_ of 1.6 μg/ml after 24 h of exposure.

**TABLE 6:** *In vitro* cell proliferation assay with HepG2 cells

Compound	EC_50_ (μg/ml)	
	24 h	72 h
ZAC989	>36	6.9 ± 0.7
ZAC307	>28	13.2 ± 0.8
EDTA	>29	>29
TPEN	1.6 ± 0.2	1.4 ± 0.8

### ZACs exhibited *in vivo* efficacy in a murine fungal kidney burden candidiasis model.

**FIG 7:**
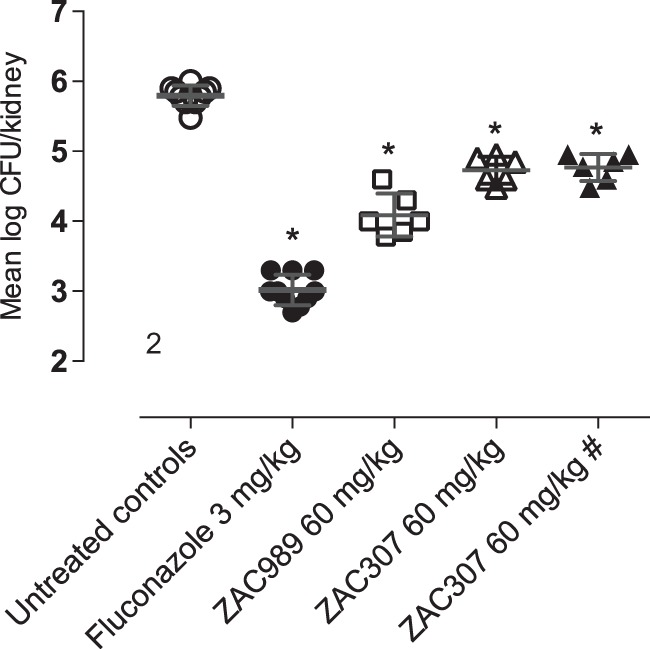
*In vivo* efficacy data for ZAC989 and ZAC307 in a 3-day candidiasis kidney burden model. Dosing with and without pretreatment (day −1) yields the same CFU reduction for ZAC307. #, no pretreatment in this arm; *, *P* < 0.05 compared to infected untreated control group by one-way analysis of variance (ANOVA).

ZAC989 and ZAC307 were investigated for *in vivo* efficacy in a fungal kidney burden model (Fig. 7; Table 7). In this model, BALB/c mice were infected intraperitoneally (i.p.) on day 0. Initially, administration of ZACs included a pretreatment 24 h prior (day −1) to infection (day 0) by the i.p. route. The mice were then treated with either ZAC for 4 days (day −1 to day 2) to maximize the likelihood of observing *in vivo* efficacy. The endpoint was mean log CFU in the kidneys of treated animals compared to the value for untreated animals. Fluconazole was chosen as a comparator compound and dosed *per os* (p.o.), and treatment with fluconazole resulted in a significant reduction in kidney burden, 2.78 log CFU/kidney (Fig. 7). The *in vivo* studies also revealed that i.p. dosing of ZAC989 at 60 mg/kg of body weight resulted in a statistically significant reduction, 1.71 log CFU/kidney, while ZAC307 administration at 60 mg/kg led to a significant reduction, 1.06 log CFU/kidney. ZAC307 yielded equal *in vivo* efficacies with and without pretreatment (Fig. 7; Table 7). We observed no adverse effects following dosing of 60 mg/kg of ZAC307, but for ZAC989, we observed lethargy lasting for 5 to 15 min after dosing.

**TABLE 7:** Fungal kidney burden candidiasis *in vivo* model

Parameter	Value with antifungal therapy^*a*^				
	None (untreated control)	Fluconazole, 3 mg/kg	ZAC989, 60 mg/kg	ZAC307, 60 mg/kg	ZAC307, 60 mg/kg, no pretreatment
Mean log CFU/kidney	5.79 ± 0.14	3.02 ± 0.22	4.09 ± 0.31	4.73 ± 0.19	4.77 ± 0.19
Mean log CFU/kidney reduction	NA	2.78*	1.71*	1.06*	1.03*

a *, *P* < 0.05 compared to infected vehicle control group, one-way ANOVA. NA, not applicable.

## DISCUSSION

During this study, we identified a new series of zinc-attenuating compounds with broad-spectrum antifungal activity *in vitro* and *in vivo* activity in a candidiasis fungal kidney burden model. The compounds ZAC307 and ZAC989 possess a characteristic arrangement of an aromatic structure with nitrogen bound in close proximity to a hydroxyl group. This structural arrangement led us to speculate whether ZAC307 and ZAC989 were metal chelators. To address this hypothesis, we synthesized ZAC623 as a control compound where the hydroxyl group is replaced with an amino group, and as expected, this compound lacked metal-chelating and antifungal properties (Fig. 1 and 3A; Table 1). The metal-chelating compounds EDTA and TPEN have previously been described as antifungal compounds (16, 17, 26); thus, they were selected as comparators in this study.

Our findings indicate that ZAC307 and ZAC989 chelate both zinc and copper, with zinc ions being most effective in reversing the growth-inhibitory effects of the ZACs (Fig. 3C and D). The ZACs have lower affinity for iron and negligible affinity for magnesium and calcium. The ZACs are less potent zinc chelators than the known zinc, copper, and iron chelators TPEN and EDTA, but they inhibit fungal growth more effectively than EDTA (Fig. 3A and B). The inhibitory effects of the ZACs can be inactivated by the addition of excess zinc in both Candida albicans and Aspergillus fumigatus, which indicates that these compounds interfere either directly or indirectly with fungal zinc homeostasis.

The first challenge that any microorganism faces in the homeostatic response to zinc deficiency is to obtain zinc from the surrounding environment. The major components of the zinc uptake system in A. fumigatus that facilitates zinc uptake from zinc-limiting media are the ZIP plasma membrane transporters ZrfA, ZrfB, and ZrfC. The ZrfA and ZrfB transporters operate mainly under acidic zinc-limiting conditions (27), although they also contribute to zinc uptake from alkaline zinc-limiting media along with ZrfC (3), which is expressed exclusively in alkaline media (24). Therefore, we reasoned that if the ZACs inhibited the intake of zinc mediated by these transporters, the growth capacity of a wild-type strain in the presence of ZACs should be reduced to the same level as that of fungal mutant strains lacking the acidic (ZrfA and ZrfB) and/or the alkaline (ZrfC) zinc transporter. The investigation of the growth capacity of a Δ*zrfC* mutant in the presence of the ZACs suggested that ZrfC plays an important role in overcoming ZAC inhibition, since deletion of *zrfC* increased the sensitivity of A. fumigatus to these compounds (AF14 versus AF54 [Fig. 5A and E]). Hence, it could be possible that the actual effect of ZACs on the Δ*zrfA* Δ*zrfB* strain was masked to some extent by the ZrfC function. As expected, the AF721 strain lacking *zrfC* did not grow under zinc-limiting conditions, and the effects of these compounds on this strain could therefore not be tested (Fig. 5C). However, we observed that ZAC989 noticeably inhibited the growth capacity of AF721 in the presence of 50 μM zinc. Thus, it is plausible that ZAC989 interfered with a zinc homeostatic process other than zinc uptake. Furthermore, the higher growth capacity of the Δ*zrfC* strain in the absence of ZACs than of the wild-type, AF48, or AF731 strain suggested that the lack of *zrfC* in AF54 may have been compensated for by the overexpression of *zrfA* and *zrfB*, as reported previously (3). On the other hand, the stronger growth inhibition of the growth capacity of the AF54 strain in the presence of ZAC307 and ZAC989 than of the wild-type, AF48, or AF731 strain suggested that the levels of expression of the *zrfA* and *zrfB* genes in AF54 were insufficient to counteract the effects of ZAC307 and ZAC989. Taken together, these results suggest that both ZAC307 and ZAC989 interfered negatively with the expression of the genes encoding zinc transporters rather than with their zinc uptake function. Indeed, gene expression analysis by RT-qPCR suggested that the antifungal effects of ZAC307 and ZAC989 were mediated through a mechanism that ultimately results in inhibition of the transcriptional activation activity of ZafA. In addition, and since ZafA activity was inactivated by zinc under physiological conditions, the effects of ZAC307 and ZAC989 on the expression of ZafA-regulated genes could be exerted either directly, upon their binding to ZafA, or indirectly by increasing the cytosolic concentration of Zn^2+^ ions that would bind to and inactivate ZafA. Although direct binding of ZAC307 or ZAC989 to ZafA was an attractive possibility, we consider it more likely that the ZACs triggered a transient rise in the cytosolic concentration of Zn^2+^ ions by favoring their releasing from cytosolic zinc ligands and/or zinc storage compartments (e.g., the vacuole). For the data presented in Fig. 4D, we observed that the ZACs promote a decrease in the available zinc levels within the internal membrane system, including the ER, in C. albicans. This decrease can be explained either by a direct competition between Zinbo-5 and ZACs within these compartments or by promotion by the ZACs of the release of zinc from the ER into the cytosol. The latter scenario could induce a higher concentration of zinc in the cytosol, and if the same situation occurs in A. fumigatus, this could explain the decrease in transcription of the ZafA-induced genes, similar to when zinc is added extracellularly. Moreover, since most intracellular eukaryotic zinc proteins bind zinc with a higher affinity than ZACs (*K_d_* values for most zinc-binding proteins are between 0.1 and 1.0 pM) (28), it is more likely that these compounds interfere with the regulation of zinc homeostasis by promoting the release of Zn^2+^ ions from intracellular storage compartments. Nevertheless, since acidification of cytoplasm can also promote zinc release from cytoplasmic zinc ligands (29), it could also be possible that the ZACs disturbed the activity of proteins involved in maintaining the pH homeostasis of the cytoplasm. In this regard, we observed that the expression level of *pmaA* was higher under zinc-replete than under zinc-limiting conditions. This finding suggests a putative interplay between the function of PmaA and the Zn^2+^ transport capacity of the fungal zinc transporters, provided that the expression level of the *pmaA* gene correlated with the H^+^-ATPase (PmaA) activity. It is known that the accumulation of zinc into the vacuole by CDF transporters (e.g., ZrcA) is mediated through a Zn^2+^/H^+^ antiporter mechanism and relies on the proton gradient generated by the V-ATPase (30). In contrast, and although it is not completely known how ZIP proteins transport zinc across the plasma membrane, it seems that it is not dependent on the proton gradient generated by the plasma membrane H^+^-ATPase but is pH dependent, such that intracellular acidification increases zinc transport, whereas extracellular acidification decreases zinc transport (31–33). In this regard, it is plausible that under the alkaline zinc-limiting conditions provided by sRPMI medium, a low PmaA activity would increase intracellular acidification to favor zinc uptake by ZIP transporters located in the plasma membrane. In contrast, a high PmaA activity under zinc-replete conditions would reduce intracellular acidification and increase extracellular acidification (e.g., to counteract the Zn^2+^-induced dissipation of the electrochemical gradient that is essential for fungal survival). The same reasoning could be applied to zinc transport by the ZrfF ZIP transporter located in the vacuolar membrane, such that the intravacuolar pH, which is kept lower than the cytosolic pH under normal conditions via V-ATPase activity, would favor the exit of Zn^2+^ ions into the cytosol. However, the unexpected finding that ZAC307 reduces *pmaA* expression suggested that it might interfere with the putative mechanism that links the regulation of zinc homeostasis with the function of PmaA.

The ZACs display broad-spectrum fungistatic activity and exhibit a low propensity for acquired resistance development compared to that of fluconazole. Additionally, they are superior antifungal agents to the nonpermeative chelator EDTA, and our RT-qPCR data suggest that the ZACs affect fungal zinc homeostasis differently from the very potent chelator TPEN. Therefore, the ZACs act distinctly from either EDTA or TPEN, both of which have previously been investigated as antifungal agents. EDTA has been evaluated as a combination treatment together with amphotericin B lipid complex (ABLC) in an invasive pulmonary aspergillosis model in immunosuppressed rats. The combination of EDTA with ABLC led to improved survival times and a lower tissue burden of A. fumigatus than with either agent alone (26). Furthermore, TPEN has been shown to significantly increase survival after 7 days compared to that with vehicle treatment in a murine model of invasive pulmonary aspergillosis (16). Moreover, administration of either of the two zinc-chelating agents phenanthroline and TPEN has been shown to lead to significant improvements in survival with concomitant reduction in fungal burden in immunosuppressed mice intranasally infected with A. fumigatus. Finally, it was shown that TPEN given in combination with caspofungin significantly increased survival times in murine models of invasive aspergillosis compared to that with either drug alone (17).

There is an unmet need for novel antifungal agents with broad-spectrum antifungal activity and low potential for resistance development for the treatment of invasive fungal infections. With potent antifungal activity both *in vitro* and *in vivo*, the ZACs fulfill these criteria, and their advancement in a drug development program is therefore warranted. Interestingly, the ZACs and EDTA did not inhibit mammalian cell proliferation considerably within the first 24 h of exposure (EC_50_ > 28 μg/ml), in contrast to TPEN (EC_50_ = 1.6 μg/ml), and even after 72 h of ZAC exposure, we observed a >12-fold selectivity index in growth inhibition between C. albicans and HepG2 cells; the index between A. fumigatus and HepG2 cells was limited. However, it should be taken into account that metal ions are crucial in every cellular system, including the host. Therefore, any intervention aiming to treat an infection through ion sequestration must deal with the delicate balance between positive and negative effects in both the pathogen and the host. The therapeutic safety window as well as whether the ZACs can induce zinc deficiency in the host still needs to be addressed. In summary, interfering with fungal zinc-dependent processes represents a promising new approach to antifungal therapy, and this series of zinc-attenuating compounds represents a potentially new class of antifungal agents.

## MATERIALS AND METHODS

### Synthesis of ZAC307, ZAC989, and ZAC623.

ZAC307 {2-[6-(dimethylamino)pyrimidin-4-yl]-5-phenyl-pyrazol-3-ol} synthesis pathway: (a) (i) *N*-methylmethanamine, TEA, 2-propanol, 0°C, 2 h, evaporate, (ii) hydrazine hydrate, reflux, 2 h, column chromatography, yield 65%; (b) ethyl 3-oxo-3-phenyl-propanoate-2-propanol, reflux, 1 h, yield: 69%, ^1^H nuclear magnetic resonance (NMR) (400 MHz; DMSO-*d_6_*, d): 13.2 (1H, bs), 8.49 (1H, d), 7.89 (2H, bd), 7.43 (3H, m), 6.92 (1H, bs), 6.13 (H, s), and 3.17 (6H, s).

ZAC989 {3-[(3*S*)-1-[6-(5-hydroxy-3-methyl-pyrazol-1-yl)pyrimidin-4-yl]pyrrolidin-3-yl]oxy-pyridine-4-carbonitrile} synthesis pathway: (a) NaH, abs. *tert*-butyl (3S)-3-hydroxypyrrolidine-1-carboxylate, THF, 0°C, 3 h, NH_4_Cl, 82%; (b) diethyl ether, conc. HCl, 0°C, quant.; (c) load onto SCX in methanol, elute with 1 M NH_3_ in MeOH, 95%; (d) {2-(6-chloropyrimidin-4-yl)-5-methyl-pyrazol-3-ol}: (6-chloropyrimidin-4-yl)hydrazine and methyl 3-oxobutanoate 2-propanol, reflux 90 min, 14%; (e) 2-(6-chloropyrimidin-4-yl)-5-methyl-pyrazol-3-ol and product from c, NMP, DIPEA, 30 min at 100°C, 89%, ^1^H NMR (400 MHz; DMSO-*d_6_*, δ): 8.84 (s, 1H), 8.46 (d, 1H), 8.42 (d, 1H), 7.77 (dd, 1H), 7.08 (bs, 1H), 5.59 (bs, 1H), 5.24 (bs, 1H), 3.95 (dd, 1H), 3.81 (bs, 2H), 3.68 (dq, 1H), 2.43 to 2.50 (m, 1H), 2.35 to 2.40 (m, 1H), 2.19 (s, 3H).

ZAC623 {3-[(3*S*)-1-[6-(5-amino-3-methyl-pyrazol-1-yl)pyrimidin-4-yl]pyrrolidin-3-yl]oxypyri-dine-4-carbonitrile} synthesis pathway: (a) *tert*-butyl-*N*-aminocarbamate, DIPEA, THF, rt → reflux, 98%; (b) pyrrolidine from ZAC989-c, DIPEA, NMP, 120 °C, 1 h, 71%; (c) (i) TFA, DCM, rt, 30 min, (ii) load onto SCX in methanol, elute with 0.5 M NH_3_ in MeOH, quant.; d, *Z*-3-amino-but-2-enenitrile, AcOH, EtOH, 80°C 4 h, 85%. ^1^H NMR (500 MHz; DMSO-*d_6_*, δ): 8.80 (s, 1H), 8.36 (d, 1H), 8.30 (d, 1H), 7.78 (dd, 1H), 6.84 (s, 1H), 6.65 (bs, 1H), 5.54 (bs, 1H), 5.21 (s, 1H), 3.81 (bs, 2H), 3.61 (bs, 2H), 2.25 to 2.46 (bs, 2H), 2.07 (s, 3H).

### Fungal isolates and growth conditions.

Fungal isolates used in this study were purchased from either the ATCC, DSMZ (Germany), or Danish National Serum Institute (SSI), with the exception of C. glabrata strain Cg003, which was a kind gift from Julius Subik, Comenius University in Bratislava, Slovak Republic. C. glabrata Cg003 is clinical isolate 3 previously used by Berila and Subik and was characterized to be resistant to fluconazole and itraconazole via overexpression of the multidrug resistance efflux pumps Cdr1p and Cdr2p (34). Aspergillus terreus isolate At070 was a kind gift from Herning Hospital, Denmark. The Aspergillus fumigatus strains AF14, AF54, AF48, AF721, and AF731 have been described previously (3, 24, 27).

Candida albicans SC5314, C. glabrata ATCC 90030 and Cg003, Candida krusei ATCC 6258, Candida parapsilosis ATCC 22019, and Candida tropicalis Ct016 were grown in Sabouraud broth (40 g/liter of d-glucose, 10 g/liter of peptone [pH 5.6]) or YPD (10 g/liter of yeast extract, 20 g/liter of Bacto peptone, 20 g/liter of glucose) liquid medium to mid-log phase, aliquoted into a final concentration of 20% (vol/vol) glycerol, and maintained as frozen stocks at −80°C. Frozen stocks of the mold isolates (A. fumigatus ATCC 13073, Aspergillus flavus ATCC 15547, A. terreus At070, *Rhizopus oryzae* ATCC 34965, and Rhizopus microsporus ATCC 66276) and *Mucor indicus* ATCC MYA-4678 were prepared by harvesting spores from 7-day-old potato glucose agar plates in phosphate-buffered saline (PBS) containing 0.1% Tween 80 and aliquoting these spores in the presence of glycerol at a final concentration of 20% (vol/vol).

SD medium without zinc (SDwoz; 1.71 g/liter of YNB-ZnSO_4_ [1541; Sunrise Science], 2% glucose, 5 g/liter of ammonium sulfate) was prepared in a glass beaker washed with 0.37% HCl and rinsed with water. The pH was adjusted to 7.0 with NaOH, and the medium was made sterile by filtration.

### Dissociation constant determination.

The dissociation constant (*K_d_*) of zinc to zinc-attenuating compounds was determined at room temperature using FluoZin-3 (F24194; Thermo Fisher Scientific), which has a *K_d_* (FluoZin-3–Zn^2+^) of 15 nM (35). Testing buffer consisted of PBS (pH 7.4) with 200 nM ZnCl_2_ and 500 nM FluoZin-3. In a microtiter plate, 98 μl of testing buffer was mixed with 2 μl of compound at a range of concentrations to determine the half-maximal inhibitory concentration (IC_50_). Testing plates were incubated for 2 min before being read at an excitation wavelength of 485 nm and an emission wavelength of 516 nm with a Fluoroskan Ascent microplate fluorometer (Thermo Fisher Scientific). The *K_d_*s were calculated using the equation *K_d_* (compound-Zn^2+^) = IC_50_/(1 + [FluoZin-3]/*K_d_*, FluoZin-3–Zn^2+^) (36).

### Potentiometric titration.

Potentiometric measurements were carried out in DMSO-water (70:30, vol/vol) at 25°C as described previously (22). Titrations were performed with a pH meter (Denver Instruments) utilizing a glass electrode with AgCl reference filled with 3.0 M KCl. The electrode was equilibrated in DMSO-water (70:30, vol/vol) for at least 1 h before use. All experiments were performed at a constant ionic strength (0.1 M NaClO_4_). Three milliliters of a solution containing 1 mM compound was titrated with 0.3 M NaOH by manual additions in 1- to 5-μl increments with magnetic stirring. The metal-ligand binding constants were obtained from titrations of the metal complex solutions prepared in a 1:2 metal-to-ligand ratio. The titration data were refined by the nonlinear least-squares refinement program Hyperquad2013 (37) to determine the deprotonation and stability constants.

### Antifungal susceptibility testing.

Antifungal susceptibility testing was carried out as described previously (38, 39), with a few modifications. Briefly, for each yeast or mold growth inhibition assay, frozen stocks of yeast cells or spores were diluted to a final concentration of 0.5 × 10^5^ to 2.5 × 10^5^ CFU/ml in sterile water. ZAC989, ZAC307, and ZAC623 were dissolved in DMSO to 10 mM stocks from which half-log serial dilutions were prepared. Growth assays were subsequently performed by pipetting 3 μl of compound dissolved in DMSO (giving a final concentration of 1.5% DMSO), 100 μl of cell-spore suspension, and 97 μl of 2× RPMI medium (20.8 g/liter of RPMI 1640 medium, 69.06 g/liter of morpholinepropanesulfonic acid [MOPS], 36 g/liter of glucose) into a microtiter plate that was incubated for 24 h (yeasts) or 48 to 72 h (molds) at 34°C. Fungal growth was determined spectrophotometrically by optical density reading of each well at a wavelength of 492 nm on a Victor X5 (Perkin-Elmer) plate reader. The MIC was defined as the lowest compound concentration that resulted in at least 50% growth inhibition for yeast, which corresponded to a prominent decrease in visible growth. For the molds, the MIC was defined as the lowest concentration of the compound that resulted in no visible growth. Standard errors between repeated experiments were generally below 5%. The growth effect of exogenous addition of various divalent metals was evaluated by performing the antifungal susceptibility assay in the presence of 5 μM ZAC989 or ZAC307 and with increasing concentrations (0.003 to 50 μM) of ZnSO_4_, CuSO_4_, (NH_4_)_2_Fe(SO_4_)_2_, MgCl_2_, and CaCl_2_. The minimal fungicidal concentration (MFC) was the minimum concentration that resulted in no CFU and was determined after the MIC by plating 5 μl of the mixture from wells with no visible growth onto YPD agar plates followed by 24 h of incubation at 30°C.

Growth capacity experiments with A. fumigatus strains AF14, AF54, AF48, AF732, and AF731 were performed in 24-well flat-bottomed tissue culture plates (35-3047; Falcon). A stock solution of 10 mM TPEN (P4413; Sigma) was prepared in pure ethanol. For these experiments, 5 mM stock solutions of ZAC307 and ZAC989 were prepared by dissolving each compound in 80% (vol/vol) ethanol. A 0.5 M stock solution of Na_2_EDTA·2H_2_O (1.08421.1000; Merck) was prepared in sterile water. A 1× stock solution (10.4 g/liter) of RPMI 1640 medium (R8755; Sigma) supplemented with 10 μM FeSO_4_·7H_2_O, 1 μM CuSO_4_·5H_2_O, and 1 μM MnCl_2_·H_2_O (sRPMI) was prepared under aseptic conditions and used as standard culture medium. In each well, 1 ml of culture medium containing sRPMI medium, Tween 20, ethanol (or the specified compound dissolved in 80% ethanol), and 10^5^ conidia was dispensed to achieve final concentrations of 0.7×, 0.05% (vol/vol), 1.2% (vol/vol), and 10^5^ conidia/ml, respectively. Plates were incubated for 44 h at 37°C in a humid atmosphere. To quantitate mycelial growth, each plate was scanned in an Agfa SnapScan 1236s scanner, and the intensity of the wells was quantified using the open-source image processing program Image J2. The data were represented and analyzed with Prism software 7.0.

### Time-kill assay.

C. albicans SC5314 cells (10^5^ CFU/ml) were incubated in 10 ml of RPMI medium at 30°C with gentle agitation (150 rpm) in the presence of EDTA (15 μM), TPEN (10 μM), ZAC989 (10 μM), or AMB (0.5 μM). At the desired time points (0, 3, 5.5, and 24 h), a 100-μl aliquot was removed for each test condition and serially diluted (10-fold) in saline (0.9% NaCl), and 30 μl of each dilution was plated on YPD agar plates. The colony count on each YPD plate was determined after incubation at 30°C for 48 h (40). C. albicans cells treated with DMSO (1%, vol/vol) served as a control.

### Resistance study.

The propensity for resistance development was investigated as also described previously (41), but with the following modifications. C. albicans was repeatedly exposed to either ZAC989 or ZAC307 in 1-ml cultures in SDwoz medium with a starting inoculum that had an optical density at 600 nm (OD_600_) of 0.007. A compound concentration that resulted in ∼90% growth inhibition was selected for these experiments (3.6 μg/ml for ZAC989, 2.8 μg/ml and 5.6 μg/ml for ZAC307, and 0.5 μg/ml and 1.0 μg/ml for fluconazole). Over a 36-day period, culture aliquots of 100 μl were periodically (every 1 to 2 days) transferred (passaged) to new culture tubes with 900 μl of fresh medium and fresh compound. The cells were incubated at 30 **°**C with gentle agitation (150 rpm), and the OD_600_ of cultures was monitored throughout the period to ensure that the numbers of cells exposed to compounds were comparable across treatments for each passage. Cells were passaged a total of 22 times, and cultures were periodically tested for antifungal susceptibility by following the protocol for antifungal susceptibility testing, as described above.

### Zinbo-5 assay.

C. albicans BWP17 was grown overnight in YPD medium at 30°C and 150 rpm. The cells were pelleted, washed in PBS buffer (D8537; Sigma) 3 times, and resuspended to an OD_600_ of 2.0. Two microliters of compound in DMSO was mixed with 100 μl of cell suspension and incubated statically for 1, 8, or 24 h at 30°C in a 96-well black plate. Thirty minutes before the end of the incubation period, 100 μl of 10 μM Zinbo-5 (sc-222425; Santa Cruz Biotechnology) in PBS buffer was added. The affinity constant of this probe for zinc is 2.2 nM (23). The plate was then read on a plate reader (FLUOstar Optima; BMG Lab Technologies) with excitation at 355 nm and emission at 485 nm. The decrease in Zinbo-5 fluorescence was calculated relative to the untreated DMSO control.

### RNA isolation from Aspergillus fumigatus.

A total of 1.5 × 10^6^ conidia of the wild-type strain (AF14) were inoculated into 20 ml of 0.7× sRPMI, 0.05% Tween 20, and 1.2% ethanol dispensed into 100-ml culture flasks pretreated with an overnight wash in 2 mM EDTA (pH 8.0) to minimize the presence of metal traces, followed by a thorough washing with ultrapure Milli-Q water. The flasks were subsequently sterilized in an oven at 180°C. The cultures were incubated for 20 h at 37°C and 200 rpm before the following were added: (i) pure ethanol to a final concentration of 1.2% (vol/vol) (as a reference for the transcription profiles of all genes under zinc-limiting conditions), (ii) 1.2% ethanol plus a solution of ZnSO_4_ to a final concentration 20 μM zinc (as a reference for the transcription profiles of all genes under zinc-replete conditions following the zinc shift), (iii) 1.2% ethanol plus a solution of EDTA to a final concentration of 500 μM, (iv) 1.2% ethanol plus a solution of TPEN to a final concentration of 10.6 μg/ml, and (v) a 5 mM solution of each test compound (ZAC307 or ZAC989) dissolved in 80% ethanol to a final concentration of 75 μM and 1.2% ethanol (which correspond to 21 μg/ml and 27 μg/ml, respectively). After compound addition, cultures were incubated for 2 h at 37°C and 200 rpm and mycelia were harvested by filtration through filter paper, washed twice with sterile waterm and snap-frozen in liquid nitrogen. After grinding of the mycelia in the presence of liquid nitrogen, total RNA was extracted using an RNeasy plant minikit (74904; Qiagen) according to the manufacturer's instructions. RNA was eluted in 50 μl of RNase-free water. RNA integrity was verified on 0.8% agarose gels stained with ethidium bromide. RNA was stored at −80°C until use.

### RT-qPCR.

Total RNA concentration and quality were determined by UV spectrometry (NanoDrop ND1000 spectrophotometer; Thermo Fisher Scientific), and all samples were brought to a final concentration of 150 ng/μl. Total RNA (1.5 μg) was treated with RQ1 DNase I (M610; Promega) and subsequently assessed by conventional PCR for the complete absence of genomic DNA (gDNA). Subsequently, 1 μg of DNase-treated RNA was reversed transcribed using SuperScript II reverse transcriptase (18064-014; Invitrogen, Thermo Fisher Scientific), with random hexamers (11034731001; Roche Diagnostics) as primers. Prior to qPCRs, cDNA samples were diluted 1:3 in water, except in the case of reactions against the 18S rRNA, for which samples were diluted 1:1,200 in water. qPCRs were performed on a Bio-Rad CFX96 instrument. A typical qPCR mixture (10 μl) contained 13.5 ng of cDNA (32 pg when the qPCR was for 18S rRNA), a specific pair of primers (150 nM final concentration), and the SYBR premix *Ex Taq* (RR420A; TaKaRa). Primers used for qPCR are listed in Table 5. For all qPCRs, 40 cycles were performed using the following cycling conditions: denaturation at 95°C for 10 s, annealing at 59°C for 20 s, and extension at 72°C for 20 s. The relative expression ratio (rER) was calculated using the threshold cycle (2^−ΔΔ*CT*^) method (42) using the expression level of the 18S rRNA as an internal reference.

### Human hepatocyte (HepG2) proliferation assay.

In each well of a 96-well tissue culture plate (GR-655180; Grenier), 10,000 human hepatocytes (HepG2) (85011430; Sigma) were plated in 200 μl of growth medium (Eagle’s minimum essential medium [M2279; Sigma]) supplemented with 2 mM l-glutamine (03-020-1B; Biological Industries), 1% nonessential amino acids (XC-E1154/100; Biosera), and 10% fetal bovine serum (BI-04-007-1A; Biological Industries), and plates were incubated overnight at 37°C and 5% CO_2_. The following day, fresh growth medium plus 2 μl of compound in DMSO was added. The plate was incubated for a further 24 h or 72 h at 37°C and 5% CO_2_. The medium was then replaced with 100 μl of freshly prepared 2,3-bis-(2-methoxy-4-nitro-5-sulfophenyl)-2H-tetrazolium-5-carboxanilide salt (XTT) sodium salt solution (0.5 mg/ml of XTT; X4251; Sigma) in RPMI 1640 (R7509; Sigma-Aldrich) with 3.83 μg/ml of phenazine methosulfate (P9625; Sigma-Aldrich) and incubated 2 to 3 h at 37°C and 5% CO_2_ (43). The color reaction was measured on Victor X5 plate reader (Perkin-Elmer) at OD_450_, and the half-maximal effective concentration (EC_50_) was calculated. Tamoxifen (85256; Sigma) was used as a positive-control compound.

### *In vivo* fungal kidney burden candidiasis model.

A murine model of systemic candidiasis was established according to a previously described method (44). BALB/c mice were infected with a 0.1-ml inoculum (1 × 10^5^ to 5 × 10^5^ CFU) of Candida albicans SC5314 cells by the intravenous route on day 0. Compounds were tested at doses of 60 mg/kg. Administration of compound was initiated 24 h prior (day −1) to infection (day 0) by the intraperitoneal route, dosing twice a day for 4 days (day −1 to day 2). ZAC307 was also evaluated with administration of the compounds given after the infection at day 0 (no pretreatment) and following dosing twice a day for 3 days (days 0 to 2). Fluconazole was used as a comparator drug. Six mice were used for each group, and the untreated control group was exposed to the vehicle alone. ZAC989 and ZAC307 were formulated by taking 60 mg of compound and adding this to 2 ml and 4 ml of 0.1 N NaOH, respectively. After mixing and sonication of the resulting solutions, 4 ml of purified water was added. The pH was adjusted to 9.0 with 0.1 N HCl solution, followed by the addition of 90 mg of NaCl. The solutions were then diluted to 10 ml and filtered through a 0.22-μm polyvinylidene difluoride (PVDF) filter. Sample collection and processing were performed as follows. Twelve hours after the last dose, all treated and untreated animals were sacrificed by cervical dislocation and kidneys were collected in 3 ml of sterile normal saline. The samples were homogenized, serially diluted, and plated on Sabouraud dextrose agar (SDA). SDA plates were incubated for 24 to 48 h at 35°C, and CFU were enumerated and reported as log CFU/kidney. The endpoint was mean log CFU of fungi in kidneys of treated animals compared to that of untreated animals. The study was conducted in conformance with an application submitted to the Committee for the Purpose of Control and Supervision of Experiments on Animals (CPCSEA), New Delhi, India, after approval from the Institutional Animal Ethics Committee (IAEC).

## References

[1] Hood MI, Skaar EP 2012 Nutritional immunity: transition metals at the pathogen-host interface. Nat Rev Microbiol 10:525–537. doi:10.1038/nrmicro2836. 22796883 PMC3875331

[2] Soares MP, Weiss G 2015 The iron age of host-microbe interactions. EMBO Rep 16:1482–1500. doi:10.15252/embr.201540558. 26474900 PMC4641501

[3] Amich J, Vicentefranqueira R, Mellado E, Ruiz-Carmuega A, Leal F, Calera JA 2014 The ZrfC alkaline zinc transporter is required for Aspergillus fumigatus virulence and its growth in the presence of the Zn/Mn-chelating protein calprotectin. Cell Microbiol 16:548–564. doi:10.1111/cmi.12238. 24245710

[4] Urban CF, Ermert D, Schmid M, Abu-Abed U, Goosmann C, Nacken W, Brinkmann V, Jungblut PR, Zychlinsky A 2009 Neutrophil extracellular traps contain calprotectin, a cytosolic protein complex involved in host defense against Candida albicans. PLoS Pathog 5:e1000639. doi:10.1371/journal.ppat.1000639. 19876394 PMC2763347

[5] Bianchi M, Niemiec MJ, Siler U, Urban CF, Reichenbach J 2011 Restoration of anti-Aspergillus defense by neutrophil extracellular traps in human chronic granulomatous disease after gene therapy is calprotectin-dependent. J Allergy Clin Immunol 127:1243–1252. doi:10.1016/j.jaci.2011.01.021. 21376380

[6] Crawford A, Wilson D 2015 Essential metals at the host-pathogen interface: nutritional immunity and micronutrient assimilation by human fungal pathogens. FEMS Yeast Res 15:fov071. doi:10.1093/femsyr/fov071. 26242402 PMC4629794

[7] Caza M, Kronstad JW 2013 Shared and distinct mechanisms of iron acquisition by bacterial and fungal pathogens of humans. Front Cell Infect Microbiol 3:80. doi:10.3389/fcimb.2013.00080. 24312900 PMC3832793

[8] Haas H 2012 Iron—a key nexus in the virulence of Aspergillus fumigatus. Front Microbiol 3:28. doi:10.3389/fmicb.2012.00028. 22347220 PMC3272694

[9] Corbin BD, Seeley EH, Raab A, Feldmann J, Miller MR, Torres VJ, Anderson KL, Dattilo BM, Dunman PM, Gerads R, Caprioli RM, Nacken W, Chazin WJ, Skaar EP 2008 Metal chelation and inhibition of bacterial growth in tissue abscesses. Science 319:962–965. doi:10.1126/science.1152449. 18276893

[10] Lulloff SJ, Hahn BL, Sohnle PG 2004 Fungal susceptibility to zinc deprivation. J Lab Clin Med 144:208–214. doi:10.1016/j.lab.2004.07.007. 15514589

[11] Moreno MA, Amich J, Vicentefranqueira R, Leal F, Calera JA 2007 Culture conditions for zinc- and pH-regulated gene expression studies in Aspergillus fumigatus. Int Microbiol 10:187–192. 18076000

[12] Staats CC, Kmetzsch L, Schrank A, Vainstein MH, Zamboni DS, De U, Paulo S, Mitchell A 2013 Fungal zinc metabolism and its connections to virulence. Front Cell Infect Microbiol 3:65. doi:10.3389/fcimb.2013.00065. 24133658 PMC3796257

[13] Broxton CN, Culotta VC 2016 SOD enzymes and microbial pathogens: surviving the oxidative storm of infection. PLoS Pathog 12:e1005295. doi:10.1371/journal.ppat.1005295. 26742105 PMC4712152

[14] Clark HL, Jhingran A, Sun Y, Vareechon C, de Jesus Carrion S, Skaar EP, Chazin WJ, Calera JA, Hohl TM, Pearlman E 2016 Zinc and manganese chelation by neutrophil S100A8/A9 (Calprotectin) limits extracellular Aspergillus fumigatus hyphal growth and corneal infection. J Immunol 196:336–344. doi:10.4049/jimmunol.1502037. 26582948 PMC4684987

[15] Santos ALS, Sodre CL, Valle R, Silva BA, Abi-chacra EA, Silva VL, Souza-Goncalves AL, Sangenito LS, Goncalves DS, Souza LOP, Palmeira VF, d'Avila-Levy CM, Kneipp LF, Kellett A, McCann M, Branquinha MH 2012 Antimicrobial action of chelating agents: repercussions on the microorganism development, virulence and pathogenesis. Curr Med Chem 19:2715–2737. doi:10.2174/092986712800609788. 22455582

[16] Hein KZ, Takahashi H, Tsumori T, Yasui Y, Nanjoh Y, Toga T 2015 Disulphide-reduced psoriasin is a human apoptosis-inducing broad-spectrum fungicide. Proc Natl Acad Sci U S A 112:13039–13044. doi:10.1073/pnas.1511197112. 26438863 PMC4620902

[17] Laskaris P, Atrouni A, Calera JA, D'Enfert C, Munier-Lehmann H, Cavaillon J-M, Latgé J-P, Ibrahim-Granet O 2016 Administration of zinc chelators improves survival of mice infected with Aspergillus fumigatus both in monotherapy and in combination with caspofungin. Antimicrob Agents Chemother 60:5631–5639. doi:10.1128/AAC.00324-16. 27401578 PMC5038287

[18] Vicentefranqueira R, Amich J, Laskaris P, Ibrahim-Granet O, Latgé JP, Toledo H, Leal F, Calera JA 2015 Targeting zinc homeostasis to combat Aspergillus fumigatus infections. Front Microbiol 6:160. doi:10.3389/fmicb.2015.00160. 25774155 PMC4343018

[19] Kjellerup L, Gordon S, Cohrt KO, Brown WD, Fuglsang AT, Winther A-ML 2017 Identification of antifungal H+-ATPase inhibitors with effect on the plasma membrane potential. Antimicrob Agents Chemother 61:e00032-17. doi:10.1128/AAC.00032-17. 28438931 PMC5487681

[20] Clausen JD, Kjellerup L, Cohrt KO, Hansen JB, Dalby-Brown W, Winther A-ML 2017 Elucidation of antimicrobial activity and mechanism of action by N-substituted carbazole derivatives. Bioorg Med Chem Lett 27:4564–4570. doi:10.1016/j.bmcl.2017.08.067. 28893470 PMC5609566

[21] Fahrni CJ, O'Halloran TV 1999 Aqueous coordination chemistry of quinoline-based fluorescence probes for the biological chemistry of zinc. J Am Chem Soc 121:11448–11458. doi:10.1021/ja992709f.

[22] Sanvar Nasir M, Fahrni CJ, Suhy DA, Kolodsick KJ, Singer CP, O'Halloran TV 1999 The chemical cell biology of zinc: structure and intracellular fluorescence of a zinc-quinolinesulfonamide complex. J Biol Inorg Chem 4:775–783. doi:10.1007/s007750050350. 10631609

[23] Taki M, Wolford JL, O'Halloran TV 2004 Emission ratiometric imaging of intracellular zinc: design of a benzoxazole fluorescent sensor and its application in two-photon microscopy. J Am Chem Soc 126:712–713. doi:10.1021/ja039073j. 14733534

[24] Amich J, Vicentefranqueira R, Leal F, Calera JA 2010 Aspergillus fumigatus survival in alkaline and extreme zinc-limiting environments relies on the induction of a zinc homeostasis system encoded by the zrfC and aspf2 genes. Eukaryot Cell 9:424–437. doi:10.1128/EC.00348-09. 20038606 PMC2837988

[25] Wilson S, Bird AJ 2016 Zinc sensing and regulation in yeast model systems. Arch Biochem Biophys 611:30–36. doi:10.1016/j.abb.2016.02.031. 26940262 PMC5010796

[26] Hachem R, Bahna P, Hanna H, Stephens LC, Hachem R, Bahna P, Hanna H, Stephens LC, Raad I 2006 EDTA as an adjunct antifungal agent for invasive pulmonary aspergillosis in a rodent model. Antimicrob Agents Chemother 50:1823–1827. doi:10.1128/AAC.50.5.1823-1827.2006. 16641455 PMC1472214

[27] Vicentefranqueira R, Moreno MA, Leal F, Calera JA 2005 The zrfA and zrfB genes of Aspergillus fumigatus encode the zinc transporter proteins of a zinc uptake system induced in an acid, zinc-depleted environment. Eukaryot Cell 4:837–848. doi:10.1128/EC.4.5.837-848.2005. 15879518 PMC1140092

[28] Maret W, Li Y 2009 Coordination dynamics of zinc in proteins. Chem Rev 109:4682–4707. doi:10.1021/cr800556u. 19728700

[29] Kiedrowski L 2014 Proton-dependent zinc release from intracellular ligands. J Neurochem 130:87–96. doi:10.1111/jnc.12712. 24606401 PMC4130388

[30] MacDiarmid CW, Milanick MA, Eide DJ 2002 Biochemical properties of vacuolar zinc transport systems of saccharomyces cerevisiae. J Biol Chem 277:39187–39194. doi:10.1074/jbc.M205052200. 12161436

[31] Colvin RA 2002 pH dependence and compartmentalization of zinc transported across plasma membrane of rat cortical neurons. Am J Physiol Cell Physiol 282:C317–C329. doi:10.1152/ajpcell.00143.2001. 11788343

[32] Lin W, Chai J, Love J, Fu D 2010 Selective electrodiffusion of zinc ions in a Zrt-, Irt-like protein, ZIPB. J Biol Chem 285:39013–39020. doi:10.1074/jbc.M110.180620. 20876577 PMC2998139

[33] Pedas P, Husted S 2009 Zinc transport mediated by barley ZIP proteins are induced by low pH. Plant Signal Behav 4:842–845. doi:10.4161/psb.4.9.9375. 19847115 PMC2802790

[34] Berila N, Subik J 2010 Molecular analysis of Candida glabrata clinical isolates. Mycopathologia 170:99–105. doi:10.1007/s11046-010-9298-1. 20232155

[35] Gee KR, Zhou ZL, Qian WJ, Kennedy R 2002 Detection and imaging of zinc secretion from pancreatic beta-cells using a new fluorescent zinc indicator. J Am Chem Soc 124:776–778. doi:10.1021/ja011774y. 11817952

[36] Cheng Y, Prusoff WH 1973 Relationship between the inhibition constant (KI) and the concentration of inhibitor which causes 50 per cent inhibition (I50) of an enzymatic reaction. Biochem Pharmacol 22:3099–3108. doi:10.1016/0006-2952(73)90196-2. 4202581

[37] Gans P, Sabatini A, Vacca A 1996 Investigation of equilibria in solution. Determination of equilibrium constants with the HYPERQUAD suite of programs. Talanta 43:1739–1753. 18966661 10.1016/0039-9140(96)01958-3

[38] Subcommittee on Antifungal Susceptibility Testing (AFST) of the ESCMID European Committee for Antimicrobial Susceptibility Testing (EUCAST). 2008 EUCAST definitive document EDef 7.1: method for the determination of broth dilution MICs of antifungal agents for fermentative yeasts. Clin Microbiol Infect 14:398–405. doi:10.1111/j.1469-0691.2007.01935.x. 18190574

[39] Subcommittee on Antifungal Susceptibility Testing of the ESCMID European Committee for Antimicrobial Susceptibility Testing. 2008 EUCAST Technical Note on the method for the determination of broth dilution minimum inhibitory concentrations of antifungal agents for conidia-forming moulds. Clin Microbiol Infect 14:982–984. doi:10.1111/j.1469-0691.2008.02086.x. 18828858

[40] Klepser ME, Ernst EJ, Lewis RE, Ernst ME, Pfaller MA 1998 Influence of test conditions on antifungal time-kill curve results: proposal for standardized methods. Antimicrob Agents Chemother 42:1207–1212. 9593151 10.1128/aac.42.5.1207 PMC105779

[41] Cowen LE, Sanglard D, Calabrese D, Sirjusingh C, Anderson JB, Kohn LM 2000 Evolution of drug resistance in experimental populations of Candida albicans. J Bacteriol 182:1515–1522. doi:10.1128/JB.182.6.1515-1522.2000. 10692355 PMC94447

[42] Livak KJ, Schmittgen TD 2001 Analysis of relative gene expression data using real-time quantitative PCR and the 2−ΔΔCT method. Methods 25:402–408. doi:10.1006/meth.2001.1262. 11846609

[43] Scudiero DA, Shoemaker RH, Paull KD, Scudiere DA, Paul KD, Monks A, Tierney S, Nofziger TH, Currens MJ, Seniff D, Boyd MR 1988 Evaluation of a soluble tetrazolium/formazan assay for cell growth and drug sensitivity in culture using human and other tumor cell lines. Cancer Res 48:4827–4833. 3409223

[44] MacCallum DM 2012 Mouse intravenous challenges models and applications. Methods Mol Biol 845:499–509. doi:10.1007/978-1-61779-539-8_35. 22328398

